# Burden of RSV hospital disease and its comorbidities in adults in Spain (2016–2023)

**DOI:** 10.1017/S0950268826102076

**Published:** 2026-10-14

**Authors:** Fernando Trigo, Jose Maria Guillen, Valentin Hernandez-Barrera, Ruth Gil, Angel Gil

**Affiliations:** 1 Medical Specialties and Public Health, Universidad Rey Juan Carlos, Spain; 2 Medical specialities & public health, Universidad Rey Juan Carlos Facultad de Ciencias de la Salud, Spain; 3 Medical Specialties and Public Health, Universidad Rey Juan Carlos - Campus de Alcorcón, Spain

**Keywords:** Hospitalizations, RSV, Burden of Disease, Spain

## Abstract

This observational descriptive study analyses the hospital burden of respiratory syncytial virus (RSV) among adults in Spain using the Minimum Basic Data Set (MBDS) from the Spanish National Hospital Data Surveillance System. Hospital admissions of adults diagnosed with non-bronchiolitis RSV infection between 2016 and 2023 were identified using ICD-10-CM codes. A total of 288,297 RSV-related hospitalizations were recorded, with a mean patient age of 76.18 years. The overall hospitalization rate was 92.43 per 100,000 inhabitants, and the mortality rate was 5.32 per 100,000. The most common severe comorbidities at admission were congestive heart failure, diabetes, and chronic obstructive pulmonary disease. Patients aged over 65 years had a significantly higher risk of in-hospital mortality (OR 1.78; 95% CI: 1.64–1.93) compared to those aged 40–64 years, increasing to 2.22 (95% CI: 2.05–2.40) in the presence of at least one severe comorbidity. Male sex was also associated with increased mortality risk (OR 1.24; 95% CI: 1.20–1.28). In conclusion, RSV represents a substantial cause of hospitalization and mortality among adults in Spain, particularly in patients over 60 years, males, and those with underlying comorbidities.

## Introduction

Respiratory syncytial virus (RSV), although well known for causing bronchiolitis and pneumonia in children, has in recent decades been recognized as a significant contributor to morbidity and mortality among adults over 60 years of age, particularly in those with underlying comorbidities [1,2].

RSV belongs to the *Paramyxoviridae* family, subfamily *Pneumoviridae*, along with human metapneumovirus. The primary mode of transmission is through respiratory droplets or self-inoculation of mucosal surfaces following contact with contaminated surfaces. In temperate climates, RSV exhibits a seasonal pattern, typically occurring from November to February [1,3].

In the adult population, symptomatic reinfections of the upper respiratory tract are common, especially among healthcare workers or individuals in close contact with children [1]. RSV has been identified as the causative agent in up to 12% of acute respiratory infections in adults [4].

The incidence of RSV infection among older adults in industrialized countries is estimated at 670 cases per 100,000 population annually, with a hospitalization rate of 100 per 100,000 population per year [5]. Mortality in older adults ranges from 6% to 8% [4], and in individuals over 75 years of age, it is comparable to that of influenza [6].

## Methods

### Study design and data source

Data for this observational descriptive study were obtained from the Minimum Basic Data Set of the Specialized Healthcare Activity Registry. This registry, compiled annually by the Ministry of Health, Social Services and Equality, aims to assess healthcare demand and morbidity, support clinical and epidemiological research, evaluate healthcare services, and facilitate the production of health statistics [7].

Using clinical codes from the International Classification of Diseases, 10th Revision, Clinical Modification (ICD-10-CM), data on hospital discharges of patients with diagnoses related to VRS across the entire Spanish territory were extracted. The study period ranged from 1 October 2016 to 30 September 2023, and was divided into seven seasons, each beginning on 1 October and ending on 30 September of the following year.

For rate calculations, population figures from the Spanish census projections for 2016–2023, obtained from the National Institute of Statistics, were used as denominators.

### Inclusion and exclusion criteria

Patients aged ≥18 years admitted to hospitals with a clinical code for RSV other than bronchiolitis at discharge, in any diagnostic position (H65 [B97.4], J06.9 [B97.4], J12.1, J20.5, J22 [B97.4], J98.9 [B97.4]), were included. *Discharge coding typically prioritizes clinical symptoms in the primary diagnostic positions. Since microbiological results often take several days to become available, RSV-related codes frequently appear in subsequent diagnostic positions. Therefore, to enhance the sensitivity of this study, we included RSV diagnoses recorded in any diagnostic position.*

Patients younger than 18 years and those with code J21.0 (bronchiolitis) were excluded, as this represents a paediatric diagnosis. Data were classified according to sex, age, and severe pre-existing comorbidities.

### Statistical analysis

The mean annual number of hospitalizations was distributed across five age groups. Annual hospitalization incidence (per 100,000 population), mortality rate (in-hospital deaths per 100,000 population), and case fatality rate (deaths among hospitalized RSV patients) were calculated.

A multivariate analysis was performed in which patients were stratified according to age and the presence of underlying clinical conditions, defining the following groups: A (40–64 years without clinical complications), B (40–64 years with clinical complications), C (≥65 years without clinical complications), and D (≥65 years with clinical complications). In addition, subgroups were created within groups B (B1–B7) and D (D1–D7) to compare the risk of in-hospital mortality associated with different severe pre-existing comorbidities: chronic obstructive pulmonary disease, congestive heart failure, coronary artery disease, pharmacologically treated diabetes, ischemic heart disease, stroke and related conditions, and chronic kidney disease, respectively. Furthermore, the relative risk of hospital admission in men compared to women was calculated for the overall population.

A significance level of *p* < 0.01 was used for statistical testing. Pearson’s chi-square test was applied to assess associations, and Poisson regression models were used to analyse significant differences in hospitalization rates by age group and sex. Statistical analyses were performed using Stata 16.1 and SPSS for Windows version 31.0. The assessment of comorbidities potentially associated with RSV was conducted using the Charlson Comorbidity Index (CCI) [8].

### Ethical considerations

Patient information was anonymized prior to analysis.

The authors assert that all procedures contributing to this work comply with the ethical standards of the relevant national and institutional committees on human experimentation and with the Helsinki Declaration of 1975, as revised in 2008.

The Rey Juan Carlos University Ethics Committee ruled that no formal ethics approval was required due to the retrospective nature of the data that were delivered to the researchers anonymously and deidentifed before the analysis, in strict compliance with the current Spanish and European legislation.

## Results

The study included 288,297 hospital admissions of adults with a non-bronchiolitis RSV code from the 2016–2017 to the 2022–2023 seasons. Of these, 127,099 (44.09%) were male and 161,198 (55.91%) were female (Table 1). The overall mean age at admission was 76.18 years and was lower in men (73.70 years) compared to women (78.13 years). Significant differences were found in the distribution of admissions by sex across all age groups, with higher rates in men up to the 84-year age group. In the ≥85-year group and in the overall study population, women predominated.

**Table 1.** Hospital admissions with a diagnosis code for RSV in Spain, seasons 2016–2023 Table 1. long description.

	Number of admissions			Hospitalization rates per 100.000 population (95% CI)		
	Male	Female	Total	Male	Female	Total
18–59 años	20,442 (16.08%)	18,539 (11.5%)	38,981 (13.52%)	18.88 (18.62–19.14)	17.36 (17.11–17.61)	18.13 (17.95–18.31)
60–64 años	8,834 (6.95%)	7,735 (4.8%)	16,569 (5.75%)	78.53 (76.89–80.17)	64.77 (63.33–66.21)	71.45 (70.36–72.54)
65–74 años	26,882 (21.15%)	22,605 (14.02%)	49,487 (17.17%)	154.1 (152.26–155.94)	114.76 (113.26–116.26)	133.24 (132.07–134.41)
75–84 años	38,246 (30.09%)	45,171 (28.02%)	83,417 (28.93%)	367.79 (364.11–371.47)	322.82 (319.85–325.79)	342 (339.68–344.32)
85 o más	32,695 (25.72%)	67,148 (41.66%)	99,843 (34.63%)	799.14 (790.51–807.77)	832.24 (825.97–838.51)	821.1 (816.03–826.17)
Total	127,099 (100%)	161,198 (100%)	288,297 (100%)	83.93 (83.47–84.39)	100.44 (99.95–100.93)	92.43 (92.09–92.77)

**Figure 1.**
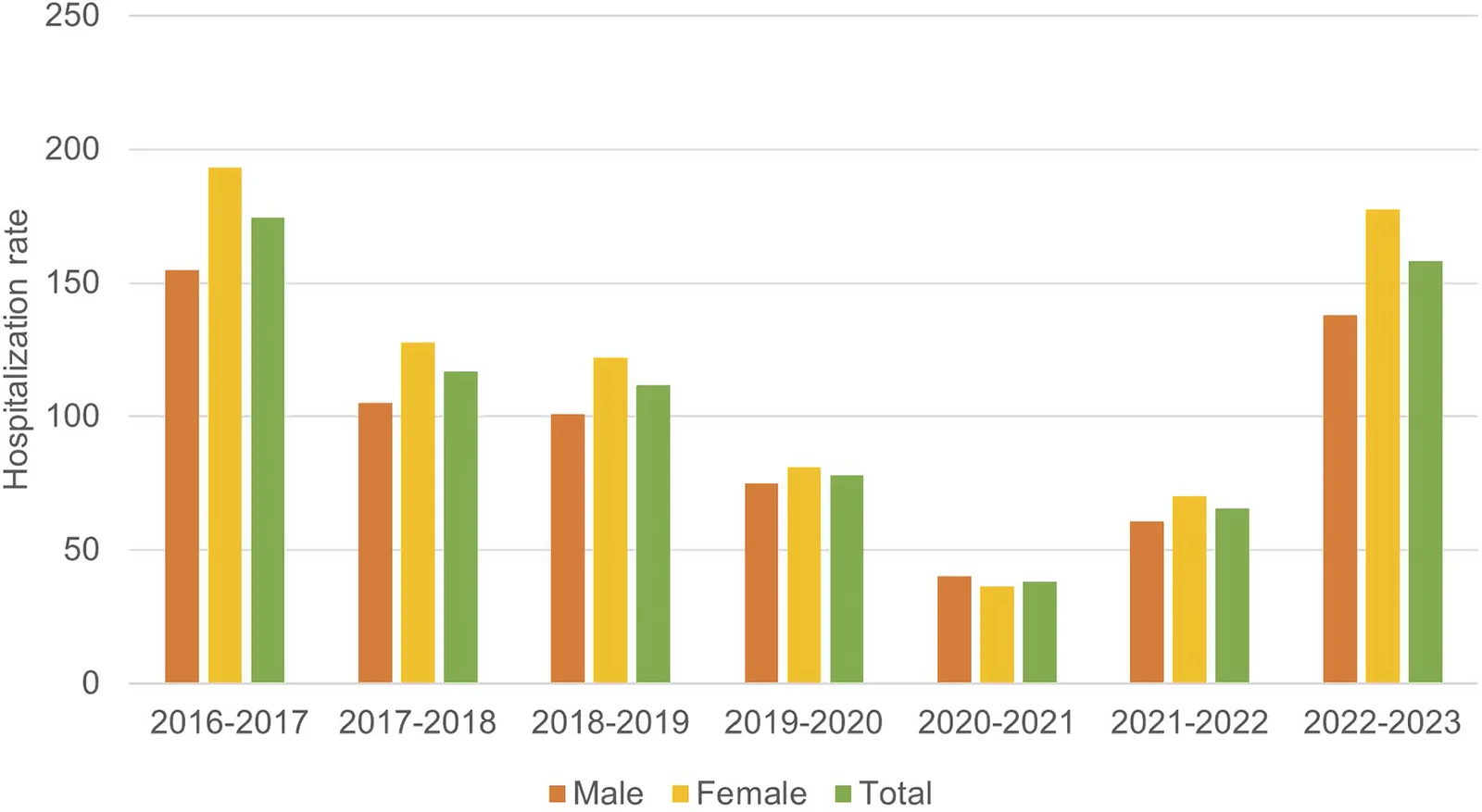
Hospitalization rate for the 2016–2017 to 2022–2023 seasons, showing distribution by sex. Figure 1. long description.

For the entire study period, the overall hospitalization rate was 92.43 admissions per 100,000 population (95% CI: 92.09–92.97). Hospitalization rates varied significantly by age (Table 1), with the lowest rate observed in the 18–59 years group (18.13 per 100,000 population; 95% CI: 17.95–18.31) and the highest in those aged ≥85 years (821.1 admissions per 100,000 population; 95% CI: 816.03–826.17). Notably, hospitalization rates also varied across seasons (Figure 1). The lowest rate was recorded during the 2020–2021 season (38.18 admissions per 100,000 population; 95% CI: 37.56–38.8), whereas the highest was observed in the 2016–2017 season (174.44 admissions per 100,000 population).

During the entire study period, a total of 16,604 in-hospital deaths were recorded among admitted patients, of which 48.74% were male and 51.26% were female.

The overall mortality rate was 5.32 deaths per 100,000 population (95% CI: 5.24–5.40), with the greatest contribution found in the ≥85-year age group, reaching 66.37 deaths per 100,000 population (95% CI: 64.92–67.82). Notably, the 18–59 years group showed a mortality rate of 0.53 deaths per 100,000 population (95% CI: 0.50–0.56), compared to 66.37 deaths per 100,000 population in those aged ≥85 years and 34.05 deaths per 100,000 population (95% CI: 33.45–34.65) in those aged ≥75 years.

**Figure 2.**
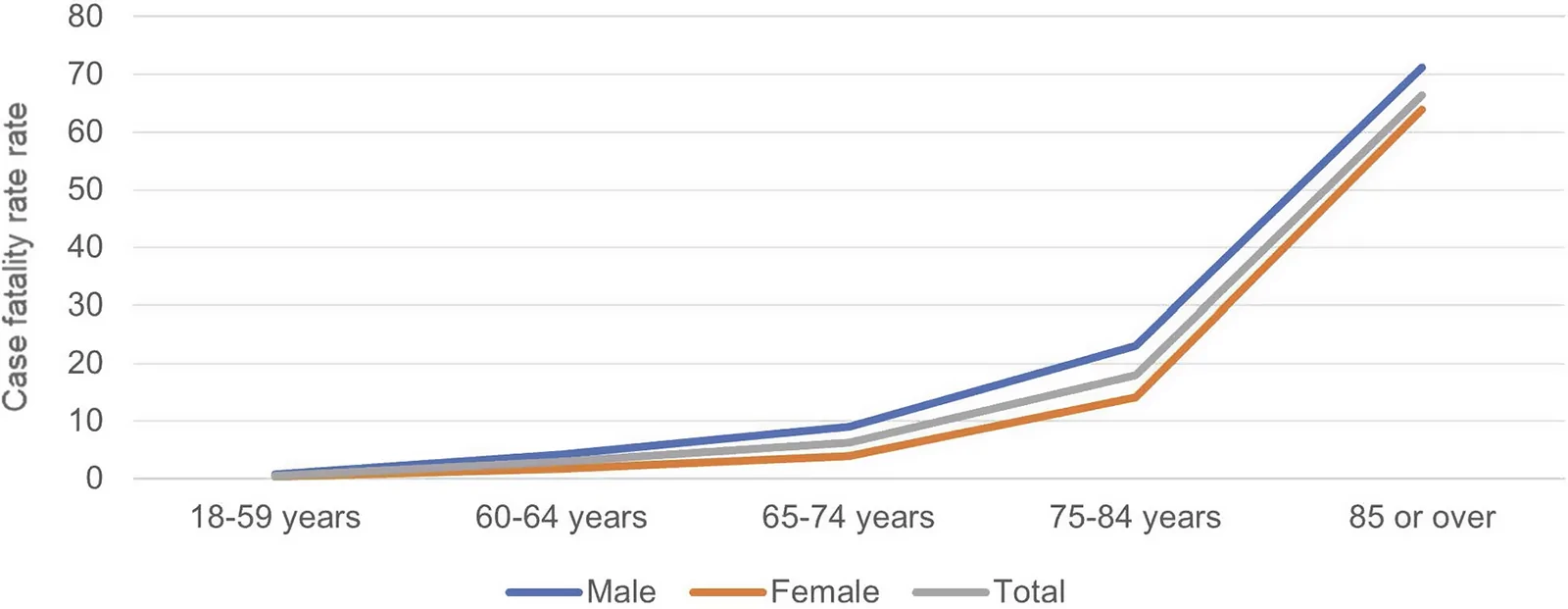
Mortality rate by age for the 2016–2023 seasons, showing distribution by sex. Figure 2. long description.

Mortality rates were higher in men. In the 65–74 years group, mortality was 8.94 deaths per 100,000 population in men compared to 3.98 in women. This difference diminished in the ≥85-year group, where mortality rates were 71.18 deaths per 100,000 population in men and 66.37 in women. Mortality rates by age and sex are shown in Figure 2.

**Figure 3.**
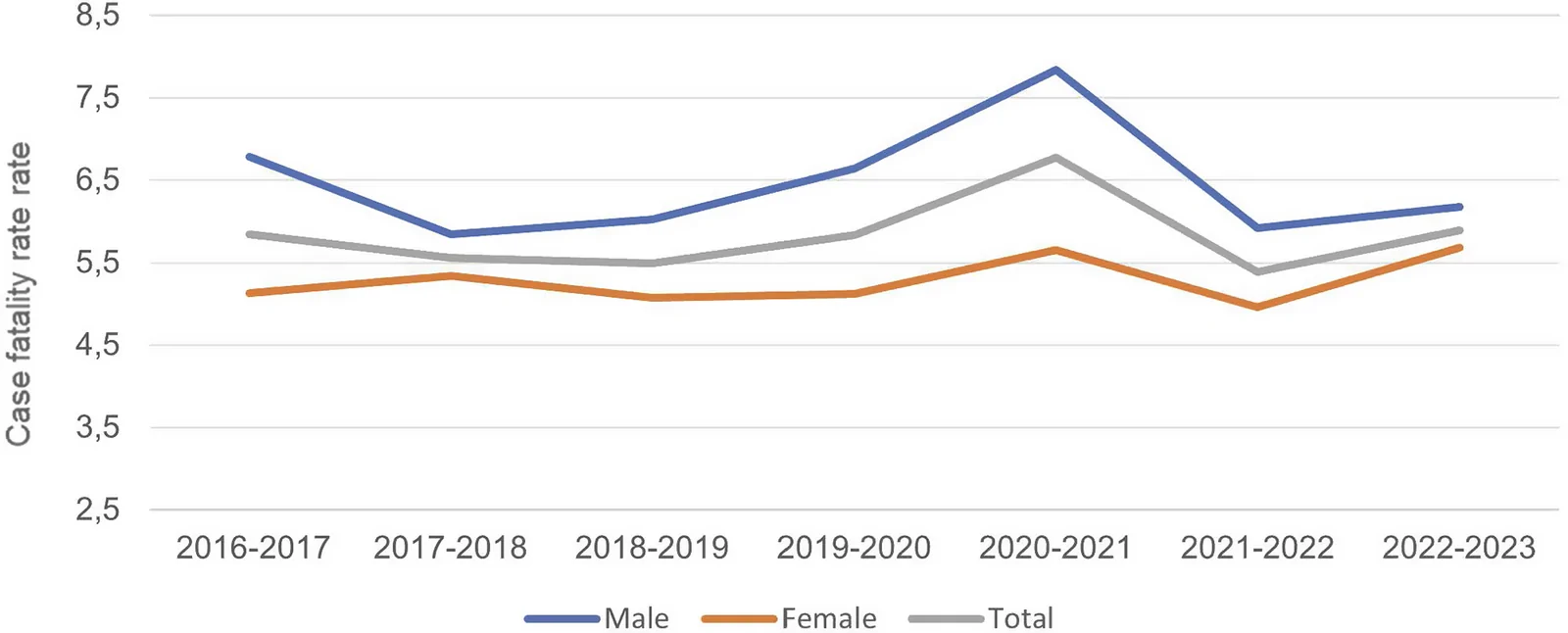
Case fatality rate by age for the 2016–2023 seasons, showing distribution by sex. Figure 3. long description.

The overall case fatality rate was 5.76% (95% CI: 5.67–5.85), reaching 8.08% (95% CI: 7.91–8.25) in the ≥85-year group. Case fatality rates by age group across different seasons are shown in Figure 3.

During the study period, the most frequent severe comorbidities among patients were, in descending order: congestive heart failure (87,939 admissions; 30.5%), pharmacologically treated diabetes (85,180; 29.55%), chronic obstructive pulmonary disease (COPD) (64,399; 22.34%), chronic kidney disease (59,766; 20.73%), ischemic heart disease (37,064; 12.86%), stroke and related conditions (19,789; 6.86%), and coronary artery disease (14,321; 4.97%).

When stratified by sex, men had significantly higher rates of diagnoses of COPD, coronary artery disease, ischemic heart disease, and stroke and related conditions, whereas women had higher rates of congestive heart failure, pharmacologically treated diabetes, and chronic kidney disease.

Furthermore, men admitted with prior COPD and coronary artery disease had significantly higher mortality compared to women with the same conditions. Similarly, women with congestive heart failure, pharmacologically treated diabetes, and chronic kidney disease had significantly higher mortality compared to men with the same conditions. No significant differences in mortality between men and women were found for ischemic heart disease and stroke and related conditions. Table 2 presents, by sex, the number of diagnoses for each severe pre-existing comorbidity at admission, as well as the number of deaths among patients with these conditions.

**Table 2.** Number of hospital admissions and deaths during hospitalization by sex among patients with prior severe comorbidity Table 2. long description.

	Number of admissions (%)				Number of deaths (%)			
	Male	Female	Total	*p*-value (male vs female)	Male	Female	Total	*p*-value (male vs female)
COPD	44,055 (34.66)	20,344 (12.62)	64,399 (22.34)	<0.001	2,308 (5.24)	816 (4.01)	3,124 (4.85)	<0.001
Congestive heart failure	32,968 (25.94)	54,971 (34.1)	87,939 (30.5)	<0.001	2,677 (8.12)	4,036 (7.34)	6,713 (7.63)	<0.001
Coronary artery disease	9,604 (7.56)	4,717 (2.93)	14,321 (4.97)	<0.001	722 (7.52)	298 (6.32)	1,020 (7.12)	0.008
Drug-treated diabetes	39,173 (30.82)	46,007 (28.54)	85,180 (29.55)	<0.001	2,341 (5.98)	2,373 (5.16)	4,714 (5.53)	<0.001
Ischaemic heart disease	22,152 (17.43)	14,912 (9.25)	37,064 (12.86)	<0.001	1,534 (6.92)	1,010 (6.77)	2,544 (6.86)	0.571
Stroke and related conditions	10,015 (7.88)	9,774 (6.06)	19,789 (6.86)	<0.001	1,070 10.68)	1,010 (10.33)	2080 (10.51)	0.422
Chronic kidney disease	26,389 (20.76)	33,377 (20.71)	59,766 (20.73)	<0.001	2092 (7.93)	2,379 (7.13)	4,471 (7.48)	<0.001

Odds ratios (OR) were calculated to compare the risk of in-hospital death in men versus women, showing a relative risk of death of 1.24 (95% CI: 1.20–1.28) for men compared to women.

Additionally, the impact of comorbidities was assessed in the 40–64-year age group, showing that patients with severe comorbidities had a 1.20-fold higher risk of death (95% CI: 1.09–1.33) compared to those without comorbidities. The risk of in-hospital death was also compared between patients aged ≥65 years without severe comorbidities and those aged 40–64 years without comorbidities, showing a higher risk in the former group (OR 1.78; 95% CI: 1.64–1.93). Finally, patients aged ≥65 years with severe comorbidities had an OR of 2.22 (95% CI: 2.05–2.40) compared to the 40–64 years group without comorbidities. These comparisons are presented in Table 3.

**Table 3.** Odds ratios comparing mortality during hospitalization risk in males and in patients aged >65 years versus those aged 40–64 years, according to the presence of severe comorbidities at admission Table 3. long description.

	Mortality risk during hospitalization by group (odds ratio, 95% CI)	
Male sex	1.24 (1.20–1.28)	
	**No severe prior comorbidity**	**Severe prior comorbidity**
40–64 years	1	1.20 (1.09–1.33)
>65 years	1.78 (1.64–1.93)	2,22 (2.05–2.40)

The impact of each comorbidity on mortality risk across different age groups was also assessed and compared with the 40–64 years group without comorbidities (Table 4). The presence of stroke or related conditions at younger ages was associated with a higher risk of in-hospital death, as observed in the 40–64 years group (OR 4.69; 95% CI: 4.05–5.43), compared to patients aged ≥65 years (OR 3.54; 95% CI: 3.23–3.84), relative to the 40–64 years group without comorbidities. Isolated COPD in the 40–64 years group appeared to be associated with a reduced risk (OR 0.72; 95% CI: 0.63–0.83), a finding not observed in patients aged ≥65 years, where it was associated with an increased risk (OR 1.84; 95% CI: 1.70–2.01), likely reflecting the effect of age. “It is also possible that individuals with a diagnosis of COPD in the 40–64 years group have a higher probability of hospitalization due to established clinical risk protocols. In contrast, those without such a diagnosis may only be admitted when presenting with a more severe clinical picture, potentially explaining the lower in-hospital mortality risk observed in this specific group.

**Table 4.** Odds ratios comparing in-hospital mortality risk in patients aged 40–64 years and ≥ 65 years for each severe comorbidity, using patients aged 40–64 years without severe comorbidities as reference Table 4. long description.

	Mortality risk during hospitalization by group and age (odds ratio, 95% CI)	
	40–64 years	65 years or more
No severe comorbidity	1	1.78 (1.64–1.93)
COPD	0.72 (0.63–0.83)	1.84 (1.70–2.01)
Congestive heart failure	1.48 (1.26–1,74)	2.67 (2.46–2.90)
Coronary artery disease	1.80 (1.43–2.26)	2.50 (2.25–2.77)
Drug-treated diabetes	1.04 (0.90–1.19)	1.94 (1.79–2.12)
Ischaemic heart disease	1.63 (1.38–1.94)	2.40 (2.20–2.62)
Stroke and related conditions	4.69 (4.05–5.43)	3.54 (3.23–3.88)
Chronic kidney disease	1.39 (1.14–1.69)	2.62 (2.41–2.84)

Regarding length of stay, the overall mean hospital stay was 8.69 days (SD: 15.98). Men had a longer mean stay (9.64 days) compared to women (7.94 days). The longest hospital stays were observed in younger age groups, particularly in the 60–64 years group (mean: 10.95 days; SD: 20.98). With increasing age, the duration of hospitalization tended to decrease, reaching a mean of 7.13 days (SD: 7.66) in patients aged ≥85 years.

## Discussion

In recent years, awareness of the impact of RSV infection in the adult population has increased considerably. Hospitalization due to RSV involves a substantial number of admissions and significant healthcare resource utilization. This is accompanied by a high mortality rate, which is concentrated among older patients and those with severe comorbidities. Furthermore, the recent introduction of RSV vaccination in adults highlights the need to better characterize disease burden, case fatality, and the influence of factors such as age, sex, and underlying clinical conditions to inform preventive strategies.

The global emergence of COVID-19 led to changes in the seasonal patterns of respiratory viruses. Consequently, international surveillance systems needed to redistribute resources, and in this context, Spain unified its Acute Respiratory Infection Surveillance System during the 2020–2021 season. RSV surveillance became integrated into the broader monitoring of acute respiratory infections alongside influenza virus and SARS-CoV-2. During the 2020–2021 season, participating Autonomous Communities observed a predominance of SARS-CoV-2, which displaced other respiratory viruses. Subsequently, at the national level, RSV circulation during the 2021–2022 season was sporadic, with an atypical epidemic wave in spring – summer 2021. Finally, RSV returned to its usual seasonal pattern in the following season [9–11].

Although the Acute Respiratory Infection Surveillance System and MBDS are conceptually different systems, the Acute Respiratory Infection Surveillance System estimates RSV-confirmed hospitalizations from population-based surveillance, whereas MBDS collects clinical diagnoses at hospital discharge within the National Health System; the data provided by the Acute Respiratory Infection Surveillance System are reasonably consistent with our findings based on MBDS. In addition, a study conducted by the Instituto de Salud Carlos III validated the comparison of hospital burden estimates in older adults between the Acute Respiratory Infection Surveillance System and MBDS for three seasons [12]. This disruption related to the pandemic and subsequent return to typical seasonality are also comparable to patterns observed in other high-income countries [13].

The reduction in hospitalizations during the COVID-19 pandemic may be explained by both hygiene measures and social distancing [14], as well as a decrease in testing and laboratory-confirmed diagnoses [15].

Across all study seasons, hospitalization rates for RSV infection varied, but consistently increased with age, reaching the highest values in patients aged >85 years. This finding is consistent with those reported in other industrialized countries [16].

Respiratory infectious diseases have traditionally been described as more frequent in men [17]. However, our study shows a higher absolute number of hospital admissions in women, who accounted for 55.91% of cases, a pattern also observed in other developed countries such as the United States [12] and in a recent study conducted in the Valencia region [18]. Nevertheless, the risk of hospitalization and case fatality were higher in men. It is also noteworthy that mortality rates in younger men were higher than in women, although this difference diminishes in older age groups. This may be partially explained by differences in the age of onset of the most common comorbidities between sexes.

The presence of severe prior comorbidities significantly increases the risk of hospitalization, as observed for conditions such as congestive heart failure and chronic obstructive pulmonary disease, in line with findings from other developed countries [19]. However, the individual impact of these comorbidities on the risk of death during hospitalization remains uncertain for many conditions [20].

In our population, RSV hospitalization is associated with high case fatality among patients aged over 60 years or those with severe preexisting comorbidities, as previously suggested by other authors [21].

The fact that confirmatory tests are not always performed for every suspected RSV case presents a challenge for data accuracy. This inconsistency, coupled with the fact that physician coding practices for RSV may fluctuate throughout the year for various reasons, creates a risk of information bias in hospital records. Therefore, our study may underestimate the true burden of disease, as reported in studies conducted in several European countries [15]. Additionally, a study from Denmark showed that RSV diagnoses in hospitalized adults were not consistently translated into ICD-10 coding [22], while a German study suggested low sensitivity of specific ICD-10 coding for RSV, contributing to underdiagnosis [23].

Patients hospitalized with RSV infection present a demographic profile similar to those hospitalized with influenza in terms of age, sex, and race [24]. However, in adults aged over 60 years, RSV hospitalization is associated with worse clinical outcomes than influenza [20]. An observational study conducted in Chicago found that patients with higher in-hospital mortality had longer delays between symptom onset and specific diagnostic testing [25]. Some authors suggest that RSV diagnosis may be delayed until influenza or COVID-19 has been excluded, potentially worsening clinical outcomes during this period [26].

In patients aged 60–89 years, mortality during hospitalization is higher when admission is due to RSV infection compared to influenza in populations where more than half of individuals had been vaccinated against influenza [27]. Similarly, in patients aged ≥65 years or those with severe comorbidities, the risk of ICU admission and the need for oxygen therapy during hospitalization are higher in RSV infections than in those caused by influenza in the same vaccinated population [28]. According to the Acute Respiratory Infection Surveillance System data, ICU admission rates in Spain were higher among hospitalized RSV cases than among hospitalized COVID-19 or influenza cases during the 2021–2022 and 2022–2023 seasons [10–11]. These findings should also be interpreted in the context of high influenza and SARS-CoV-2 vaccination coverage during the study period.

A recent study by Wee et al. [29] showed that one in ten patients hospitalized with RSV experienced a cardiovascular event during their hospital stay. Furthermore, the risk of cardiovascular events in these patients was higher than in those hospitalized with COVID-19, both in vaccinated and unvaccinated individuals, and comparable to that observed in influenza cases. It also demonstrated that the probability of acute cardiovascular events during hospitalization is similar to that of influenza. Given the close association observed between cardiovascular disease and RSV hospitalization, the potential preventive role of RSV vaccination in at-risk populations has been suggested, similar to other respiratory infections such as influenza [30].

It is important to note that during the study period, between 55% and 69% of individuals aged ≥65 years were vaccinated against influenza, whereas RSV vaccines were not yet available [31]. With the eventual introduction of RSV vaccination into adult immunization schedules, a reduction in hospitalization incidence or improved outcomes is expected, as previously observed with influenza and SARS-CoV-2 vaccination. Therefore, further studies in adults following the implementation of RSV vaccination are a priority [32].

This study has several limitations that should be acknowledged. First, the use of the MBDS administrative database may involve a degree of misrepresentation regarding the actual clinical impact of RSV on hospitalization and mortality. A significant portion of the disease burden is likely underestimated due to the problematic codification of RSV cases without systematic microbiological confirmation. In adults, it has been reported that RSV codification using ICD-10-CM codes without laboratory evidence has low sensitivity [23], meaning the data presented in this work probably represent only a fraction of the true reality. Furthermore, the lack of a uniform codification protocol across all hospitals [22] likely influenced the availability and consistency of RSV-related records within the national surveillance system. These variations in hospital coding systems and physician behaviours – which may fluctuate depending on the season or institutional practices – introduce a potential information bias that further contributes to the underestimation of the true burden of RSV disease in Spain.

*RSV represents a major cause of hospital burden among adults in Spain, particularly among older individuals and those with severe underlying comorbidities. Although women accounted for a higher number of hospital admissions, male patients experienced higher in-hospital mortality. Furthermore, the presence of severe underlying comorbidities increased the risk of death during hospitalization among patients over 60 years of age. These findings support the need to develop targeted preventive strategies for populations at increased risk of severe RSV outcomes.*

## Data Availability

The data that support the findings of this study are available from the Spanish Ministry of Health. Restrictions apply to the availability of these data, which were used under license for this study.

## Long descriptions

### Table 1. Long description

The table is organized into seven columns: Age Group, Number of Admissions (Male, Female, Total), and Hospitalization rates per 100,000 population with 95 percent C I (Male, Female, Total).

* 18 to 59 years: 38,981 total admissions (13.52 percent) with a total rate of 18.13.

* 60 to 64 years: 16,569 total admissions (5.75 percent) with a total rate of 71.45.

* 65 to 74 years: 49,487 total admissions (17.17 percent) with a total rate of 133.24.

* 75 to 84 years: 83,417 total admissions (28.93 percent) with a total rate of 342.

* 85 years or more: 99,843 total admissions (34.63 percent) with the highest total rate of 821.1. In this group, female admissions (67,148) significantly outnumber male admissions (32,695).

* Total: 288,297 admissions (127,099 male and 161,198 female) with an overall hospitalization rate of 92.43 per 100,000 population.

Navigate back to Table 1..

### Figure 1. Long description

The Y-axis is labeled Hospitalization rate with increments of 50 from 0 to 250. The X-axis lists seven seasons. Each season contains three bars: orange for Male, yellow for Female, and green for Total.

* 2016-2017: Female rate is highest at approximately 195, followed by Total at 175 and Male at 155.

* 2017-2018: Rates decrease with Female at 128, Total at 118, and Male at 105.

* 2018-2019: Rates remain stable with Female at 122, Total at 112, and Male at 101.

* 2019-2020: A significant drop occurs with Female at 81, Total at 78, and Male at 75.

* 2020-2021: The lowest point in the series with Male at 40, Total at 38, and Female at 36.

* 2021-2022: Rates begin to rise with Female at 70, Total at 66, and Male at 61.

* 2022-2023: A sharp increase with Female at 178, Total at 158, and Male at 138.

Overall, the Female rate is consistently higher than the Male rate in every season except 2020-2021.

Navigate back to Figure 1..

### Figure 2. Long description

The X axis represents five age categories: 18-59 years, 60-64 years, 65-74 years, 75-84 years, and 85 or over. The Y axis represents the Case fatality rate rate with a scale from 0 to 80 in increments of 10. Three lines are plotted: a blue line for Male, an orange line for Female, and a grey line for Total.

All three lines show an exponential increase as age increases.

* For the 18-59 years group, all rates are near zero.

* In the 60-64 years group, the Male rate begins to rise slightly above the others.

* In the 65-74 years group, the Male rate is approximately 10, the Total is approximately 6, and the Female rate is approximately 4.

* In the 75-84 years group, there is a sharper upward inflection: Male is at 23, Total is at 18, and Female is at 14.

* In the final category, 85 or over, the rates peak significantly: Male reaches approximately 71, Total reaches approximately 67, and Female reaches approximately 64.

Throughout the entire age range, the Male fatality rate remains consistently higher than the Female rate, with the Total line positioned between them.

Navigate back to Figure 2..

### Figure 3. Long description

The x-axis lists seven annual seasons from 2016-2017 to 2022-2023. The y-axis represents the Case fatality rate rate, ranging from 2.5 to 8.5 in increments of 1.0. Three lines are plotted: a blue line for Male, an orange line for Female, and a grey line for Total.

Throughout the entire period, the Male line remains the highest, the Female line the lowest, and the Total line sits between them.

* 2016-2017: Male is at approximately 6.8, Total at 5.8, and Female at 5.1.

* 2017-2018 to 2019-2020: All lines show a slight dip followed by a gradual rise. By 2019-2020, Male reaches 6.6, Total is at 5.8, and Female is at 5.1.

* 2020-2021: All three lines reach their peak. Male peaks at approximately 7.8, Total at 6.8, and Female at 5.7.

* 2021-2022: A sharp decline occurs for all groups. Male drops to 5.9, Total to 5.4, and Female to 5.0.

* 2022-2023: All lines show a slight upward trend, ending with Male at 6.2, Total at 5.9, and Female at 5.7.

Navigate back to Figure 3..

### Table 2. Long description

The table is organized into two primary sections: Number of admissions (percentage) and Number of deaths (percentage). Each section is subdivided into Male, Female, Total, and p-value (male vs female).

* C O P D: Admissions: Male 44,055 (34.66 percent), Female 20,344 (12.62 percent), Total 64,399 (22.34 percent), p-value less than 0.001. Deaths: Male 2,308 (5.24 percent), Female 816 (4.01 percent), Total 3,124 (4.85 percent), p-value less than 0.001.

* Congestive heart failure: Admissions: Male 32,968 (25.94 percent), Female 54,971 (34.1 percent), Total 87,939 (30.5 percent), p-value less than 0.001. Deaths: Male 2,677 (8.12 percent), Female 4,036 (7.34 percent), Total 6,713 (7.63 percent), p-value less than 0.001.

* Coronary artery disease: Admissions: Male 9,604 (7.56 percent), Female 4,717 (2.93 percent), Total 14,321 (4.97 percent), p-value less than 0.001. Deaths: Male 722 (7.52 percent), Female 298 (6.32 percent), Total 1,020 (7.12 percent), p-value 0.008.

* Drug-treated diabetes: Admissions: Male 39,173 (30.82 percent), Female 46,007 (28.54 percent), Total 85,180 (29.55 percent), p-value less than 0.001. Deaths: Male 2,341 (5.98 percent), Female 2,373 (5.16 percent), Total 4,714 (5.53 percent), p-value less than 0.001.

* Ischaemic heart disease: Admissions: Male 22,152 (17.43 percent), Female 14,912 (9.25 percent), Total 37,064 (12.86 percent), p-value less than 0.001. Deaths: Male 1,534 (6.92 percent), Female 1,010 (6.77 percent), Total 2,544 (6.86 percent), p-value 0.571.

* Stroke and related conditions: Admissions: Male 10,015 (7.88 percent), Female 9,774 (6.06 percent), Total 19,789 (6.86 percent), p-value less than 0.001. Deaths: Male 1,070 (10.68 percent), Female 1,010 (10.33 percent), Total 2,080 (10.51 percent), p-value 0.422.

* Chronic kidney disease: Admissions: Male 26,389 (20.76 percent), Female 33,377 (20.71 percent), Total 59,766 (20.73 percent), p-value less than 0.001. Deaths: Male 2,092 (7.93 percent), Female 2,379 (7.13 percent), Total 4,471 (7.48 percent), p-value less than 0.001.

Navigate back to Table 2..

### Table 3. Long description

The table presents mortality risk during hospitalization expressed as odds ratios with 95 percent C I.

Row 1: Male sex has an overall odds ratio of 1.24 with a C I of 1.20 to 1.28.

The subsequent section compares age groups against a baseline of 40 to 64 years across two categories: No severe prior comorbidity and Severe prior comorbidity.

Row 2 (40 to 64 years):

- No severe prior comorbidity: 1 (Reference group).

- Severe prior comorbidity: 1.20 with a C I of 1.09 to 1.33.

Row 3 (Greater than 65 years):

- No severe prior comorbidity: 1.78 with a C I of 1.64 to 1.93.

- Severe prior comorbidity: 2.22 with a C I of 2.05 to 2.40.

Navigate back to Table 3..

### Table 4. Long description

The table contains three columns: Comorbidity, 40 to 64 years, and 65 years or more. Data is presented as odds ratios with 95 percent C I in brackets.

* No severe comorbidity: 40 to 64 years is the reference at 1. 65 years or more is 1.78 [1.64 to 1.93].

* C O P D: 40 to 64 years is 0.72 [0.63 to 0.83]. 65 years or more is 1.84 [1.70 to 2.01].

* Congestive heart failure: 40 to 64 years is 1.48 [1.26 to 1.74]. 65 years or more is 2.67 [2.46 to 2.90].

* Coronary artery disease: 40 to 64 years is 1.80 [1.43 to 2.26]. 65 years or more is 2.50 [2.25 to 2.77].

* Drug-treated diabetes: 40 to 64 years is 1.04 [0.90 to 1.19]. 65 years or more is 1.94 [1.79 to 2.12].

* Ischaemic heart disease: 40 to 64 years is 1.63 [1.38 to 1.94]. 65 years or more is 2.40 [2.20 to 2.62].

* Stroke and related conditions: 40 to 64 years is 4.69 [4.05 to 5.43]. 65 years or more is 3.54 [3.23 to 3.88].

* Chronic kidney disease: 40 to 64 years is 1.39 [1.14 to 1.69]. 65 years or more is 2.62 [2.41 to 2.84].

Navigate back to Table 4..
